# Supplementary figures and images for: The regenerative period of somatosensory nerves is closed by a DCC signaling axis
Source: PLoS Genet. 2026 Feb 2;22(2):e1012033. doi: 10.1371/journal.pgen.1012033 (PMC12885379; doi:10.1371/journal.pgen.1012033)

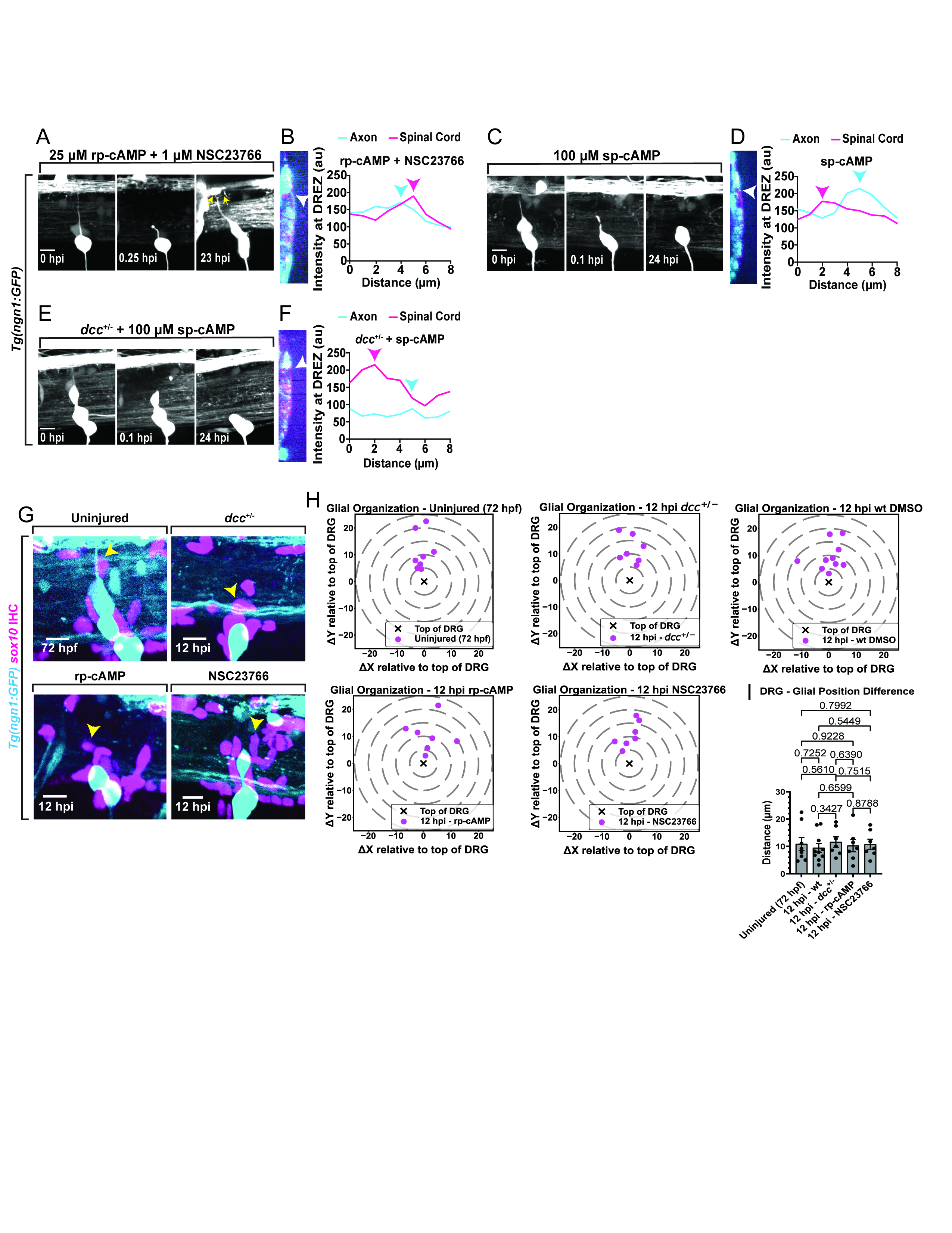

Supplement: S1 Fig — A, C and E) Max projection images of Tg(ngn1:GFP) DRG and central axons pre-axotomy (0 hpi), post-axotomy (0.1-0.25 hpi) and 23–24 hpi. Scale bar = 10 µm. Yellow arrows point to bifurcated axons. The treatment conditions are listed above images. B, D and F) DRG axons’ orthogonal images and orthogonal displacement quantifications. White arrowheads indicate the axons’ location in the orthogonal images. G) Representative images of Sox10 staining (IHC) labelling glial nuclei (pink) in 3 dpf Tg(ngn1:GFP) animals. These images represent animals that were fixed: Uninjured at 72 hpf (WT), 12 hpi in dcc+/– animals treated with 1% DMSO, 12 hpi treated with rp-cAMP (WT), and 12 hpi treated with NSC23766 (WT). Scale bars = 10 µm. Yellow arrowheads point to the dorsal-most glial nuclei. H) Glial Organization target graphs of the distance between the dorsal border of the DRG (black X) and the center of the dorsal-most glial nuclei across each biological replicate (pink circles) in each group. I) DRG to Glial Nucleus Position Difference graph display the distances between the DRG and dorsal-most glial nuclei (± SEM) per group. The average distances ± SEM are: Uninjured (72 hpf) 10.86 ± 2.404 µm (n = 8), 12 hpi WT (DMSO) 9.472 ± 1.614 µm (n = 10), 12 hpi dcc+/– (DMSO) 11.59 ± 1.912 µm (n = 7), 12 hpi rp-cAMP 10.26 ± 2.256 µm (n = 7), 12 hpi NSC23766 10.77 ± 1.803 µm (n = 7). Kruskal-Wallis One-Way ANOVA (p = 0.05). Raw data information for this figure can be found in S1 Data. (TIF) [file pgen.1012033.s002.tif]

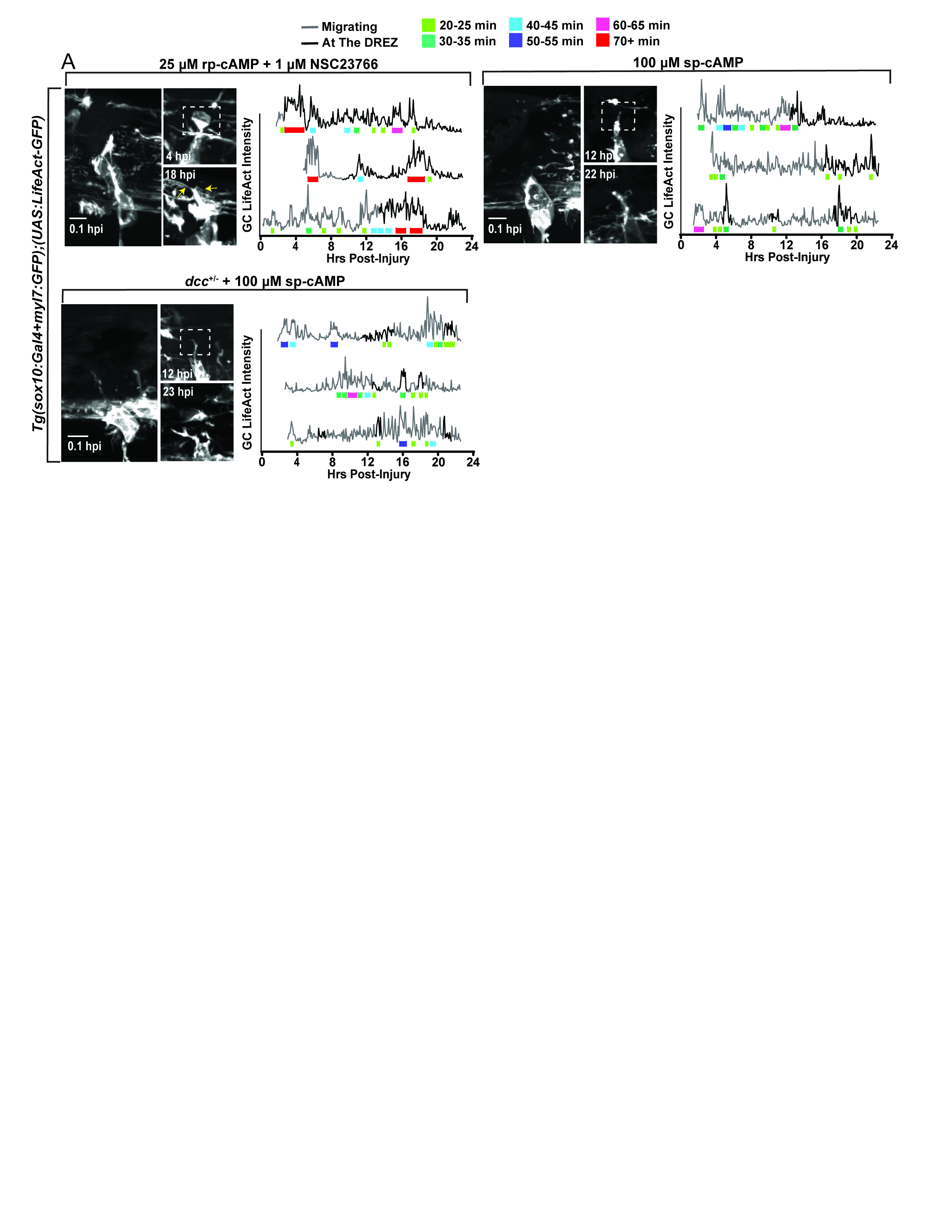

Supplement: S2 Fig — A) Max projections of Tg(sox10:Gal4 + myl7:GFP);(UAS:LifeAct-GFP) DRG post-axotomy (0.1 hpi) and growth cones during 24 hpi timelapses. White dashed boxes indicate the growth cone positioned at the DREZ, yellow arrows point to bifurcated axons. Treatment conditions for each are listed at the top of each panel. Quantifications of growth cone (GC) LifeAct intensity measurements in 3 regenerative growth cones under antagonistic treatment (rp-cAMP + NSC23766) and agonistic treatments (sp-cAMP in WT and dcc+/– animals) over the 22–24 hpi timelapses. The grey portions of these line graphs indicate when the growth cone is navigating, and the black portions indicate when the growth cone was at the DREZ. Colored brackets under peaks of LifeAct fluorescence represent their duration. See key above. Raw data information for this figure can be found in S1 Data. (TIF) [file pgen.1012033.s003.tif]

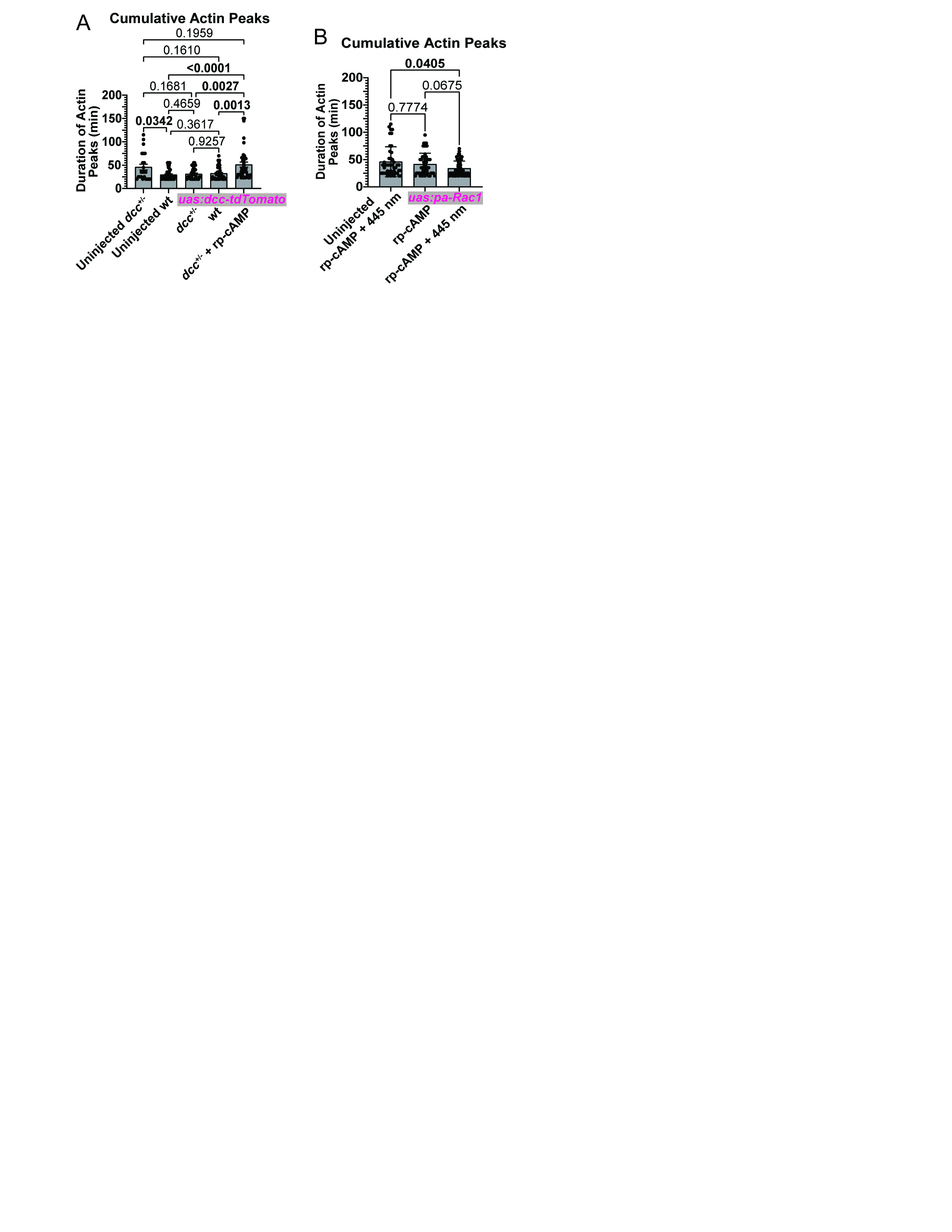

Supplement: S3 Fig — A) Quantification of LifeAct peak durations (20 min or more) occurring in the growth cone throughout the entire 22–24 hour timelapse in DCC-tdTomato expressing DRG and uninjected controls. Uninjected dcc+/– n = 3, Uninjected wt n = 4, DCC-tdTomato+ dcc+/– n = 4, DCC-tdTomato+ wt siblings n = 4, DCC-tdTomato+ dcc+/– animals in rp-cAMP treatment n = 5. B) Quantification of LifeAct peak durations (20 min or more) occurring in the growth cone throughout the entire 22–24 hour timelapse in pa-Rac1-mCherry+ DRG and uninjected controls. Uninjected rp-cAMP exposed to 445 nm light n = 6, pa-Rac1 expressing rp-cAMP treated unexposed to 445 nm light n = 6, pa-Rac1 expressing rp-cAMP treated exposed to 445 nm light n = 7. For (A) and (B), comparisons between groups were made with Kruskal-Wallis One-Way ANOVA tests. Raw data information for this figure can be found in S1 Data. (TIF) [file pgen.1012033.s004.tif]

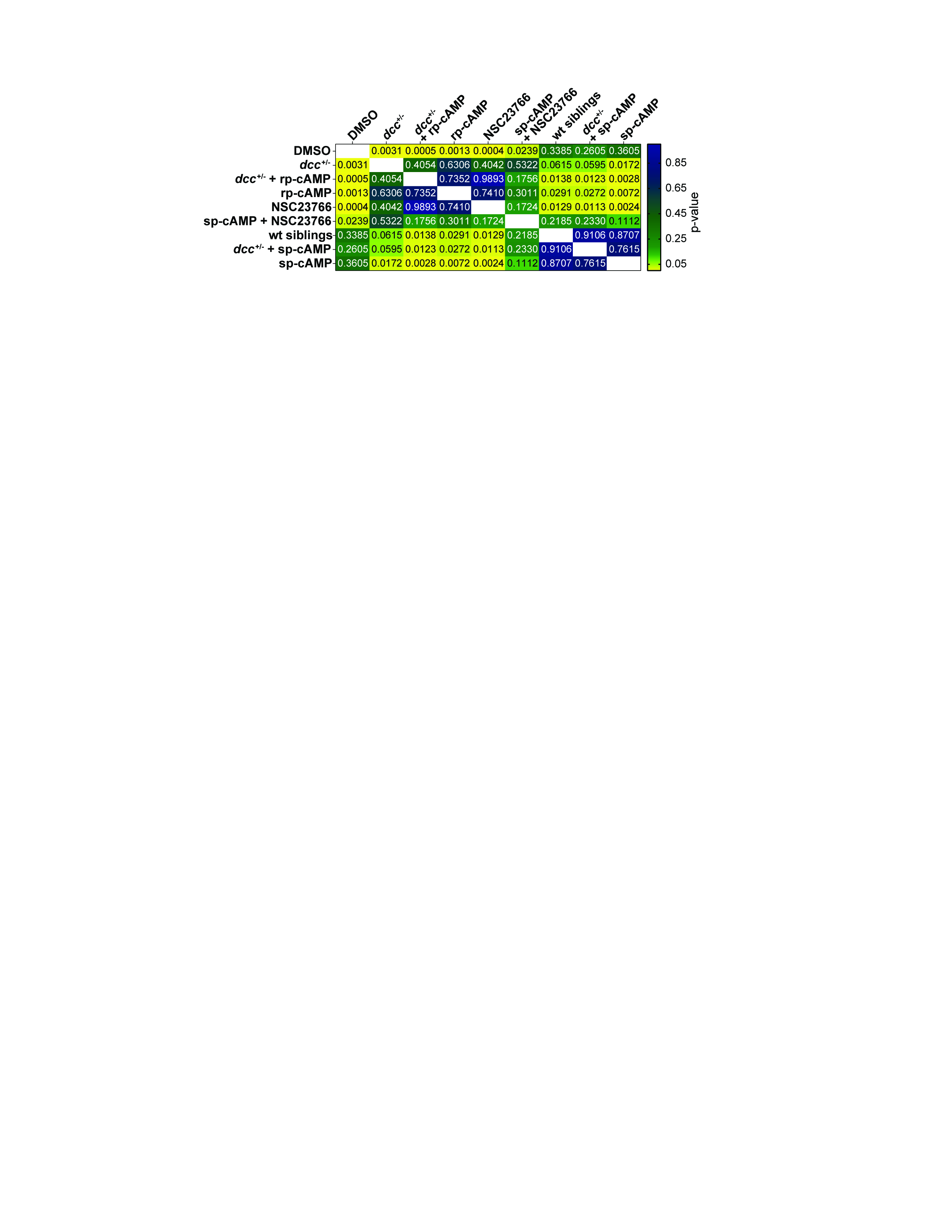

Supplement: S4 Fig — The raw data information for this figure can be found in S1 Data. (TIF) [file pgen.1012033.s005.tif]
