# Supplementary material for: The regenerative period of somatosensory nerves is closed by a DCC signaling axis
Source: PLoS Genet. 2026 Feb 2;22(2):e1012033. doi: 10.1371/journal.pgen.1012033 (PMC12885379; doi:10.1371/journal.pgen.1012033)
Supplement: Attachment — Submitted filename: Response to Reviewers Hammer et al. v2.pdf [file pgen.1012033.s022.pdf]

RESPONSE TO REVIEWERS

We would like to thank the reviewers and editors for their thoughtful comments. We also thank the reviewers for describing the study as important, well done, and novel. We have addressed most of the comments with new experiments or analysis. The remaining comments were addressed with text changes to the manuscript. The main experimental changes to the manuscript include:

1. New control experiments with photoablation to surrounding tissue
2. New quantification of regeneration after cell-specific manipulations
3. New experiments to reveal the role of DCC/cAMP/Rac1 in glial organization

New data is now available in Figures 2, 4, 6. Figures 1,2,3,5,6 have all been modified to address the reviewer concerns.

The new version of the manuscript has improved as a result of the reviewers' thoughtful comments. Below, we provide the response to each reviewer comment. The resubmission also includes a file with marked changes to the manuscript.

Additional Editor Comments:

The academic editor has obtained 3 reviews, which are included below. The editor requests that you constructively address the concerns of the reviewers in a revised manuscript. In particular, the following issues should be addressed.
1. The concept of a regenerative window closing/opening versus a delay in closing should be explained. If a window manipulated with DCC/cAMP/Rac1 "reopened" it means that it was initially closed. However, there could be a delay or a decline in regenerative potential. Data supporting regenerative potential after 3 dpf could be useful to clarify this point.

Thank you for highlighting this concept. As requested by the reviewers, we tested if the regenerative period can be re-opened beyond 3 dpf (when the regenerative window has closed) by manipulating DCC/cAMP/Rac1. This experiment required an experimental paradigm where we could temporally control the manipulation, thus we utilized pharmacological treatments rather than genetic mutation of *dcc,* as it could leave the regenerative period at least partially open. We addressed this concept by performing single central axon injuries in 5 dpf animals co-treated with rp-cAMP and NSC23766 (the cAMP and Rac1 antagonists). The rationale for co-treating was that the double manipulations generated the highest regeneration rate at 3 dpf (Figure 4B) and ensured that any regenerative window would be fully closed prior to us testing if we can re-open it. Our new results demonstrate that 30% of central axons were able to regenerate across the DREZ in co-treated 5 dpf animals compared to 0% in the untreated condition (Figure 4I-K). These findings provide strong evidence that antagonistic manipulation of the DCC signaling cascade not only extends the regenerative period but re-opens it. We feel this not only solidifies our claims but strengthens the therapeutic potential of such treatment.

2. Provide more evidence regarding the damage caused by photoablation by doing and showing "mock" control ablations outside the axons.
3. The localization of glia should be characterized and the direct involvement of glia should be further characterized by doing glia ablation and comparing to "mock" ablation outside the glia.

We appreciate these additional controls that were recommended by the reviewers. We addressed issues 2 and 3 – how photoablation around/outside the axon and glia affects glial re-organization – by performing mock glial ablations and mock axotomies targeted adjacent to the DRG axon and ensheathing glia in 3 dpf animals (Figure 2A). After the mock ablations and mock axotomy, timelapse imaging and quantification of glial re-organization was completed (Figure 2B & 2C). We tracked glial migrations 4 hours post mock ablation/axotomy since this was the average time in which we observed central axons typically began to re-extend when central axon axotomies were performed in 3 dpf animals. This allowed us to measure glial re-organization in comparable time frames with the previous experiments. Mock ablation did not induce glial migration to mock ablation sites nor did we detect a difference in the spatial migration distances of glial nuclei when compared to axotomized 3 dpf animals (Figure 2D). However, glial migration distances in 2 dpf animals were still significantly less than mock injured animals (Figure 2D).This experiment and the results demonstrate that photoablation, in general, does not induce glial re-organization but rather that dorsal migration is an innate developmental process that occurs ~3 dpf, directly coinciding with regenerative period closure.

We also performed mock glia ablations (outside of the glia) prior to central axon axotomy to confirm that glial ablation rather than photodamage in general are increasing regeneration rates. Mock glia ablations (prior to central axon axotomy) did not improve central axon regeneration rates, further supporting that glia have a role in regenerative period closure and that glia ablations do have pro-regenerative effects on the central axon in 3 dpf animals (Figure 2F).

In addition to these added experiments and analyses, we also performed central axon injuries in 3 dpf animals to see if in antagonistic manipulation (heterozygous *dcc* mutation, treatment with rp-cAMP or NSC23766) altered glial organization (Figure 4L, Figure S1G-I). The effects of these manipulations were analyzed 12 hpi and compared to uninjured 3 dpf animals and wildtype untreated injured animals 12 hpi. We concluded that antagonistic manipulations did not affect glial re-organization (Figure 4L, Figure S1I), which suggests that the pro-regenerative effects of these manipulations are likely acting upon the regenerative axon/growth cone.

Collectively, these additional experiments support our previous hypotheses and strengthen our initial findings.

4. Study whether DCC and Rac1 manipulation alter regenerative potential.

We appreciate this question as it really gets to the core of the study. While we had demonstrated that: 1) the formation of stable invadopodia at the DREZ are necessary for successful regeneration, and 2) that DCC and Rac1 were regulators of this process from our cell-specific manipulations; we had not addressed if these cell-specific manipulations extended

these effects on regenerative potential directly. Our ability to test this was limited by the available fluorescent markers used in this study. For instance, our re-entry analyses (orthogonal displacement quantifications) utilizes both *ngn1*:GFP^{+} DRG/central axons and *gfap*:mCherry^{+} spinal cord boundary to score if the axon had crossed into the CNS. Therefore, expression of *dcc*-tdTomato and pa-Rac1:mCherry in DRG cells would be nearly impossible to detect due to the robust fluorescence of *gfap*:mCherry in the transgenic background. Further, if it were detectable, it would be challenging to distinguish between those fused proteins and the mCherry^{+} glial limitans.

In order to address this question, we re-evaluated our previous cell-specific manipulation experiments of DCC and Rac1 in the LifeAct movies to score if regeneration was also affected. This scored the cell-autonomous effects on regenerative potential by quantifying if regenerative axons maintained their position at the DREZ by the end of the 24 hpi timelapse, reminiscent of regeneration, or if they subsequently retracted, indicative of regeneration failure. We observed that DCC-tdTomato^{+} DRG of *dcc*$^{+/-}$ animals treated with rp-cAMP had a marked increase in axons that remained positioned at the DREZ compared to DCC-tdTomato^{+} DRG of *dcc*$^{+/-}$ animals not treated with the downstream antagonist, which had significantly more axons that had subsequently retracted (Figure 6E). This indicates that cell-specific overexpression of DCC has direct, negative influences on not only invadopodia (Figure 6D) but regenerative potential. This not only recapitulated the wildtype phenotype – destabilized invadopodia – in *dcc*$^{+/-}$ animals (Figure 6D) but increased the likelihood of retraction (Figure 6E). These results also further support our previous claims that the stabilizing effects of rp-cAMP treatment on invadopodia dynamics have positive effects on regeneration. While this analysis does not definitively demonstrate regeneration through the glial limitans boundary, as we cannot measure re-entry directly, we are confident that it further supports our claims that DCC signaling normally limits regeneration cell-autonomously.

We repeated this regenerative potential analysis for pa-Rac1 injected animals treated with rp-cAMP that were either exposed to the activating 445 nm light or not, and compared these to rp-cAMP treated uninjected controls (that were exposed to 445 nm light). We observed that rp-cAMP treatment in uninjected controls and unexposed (and thus unactivated) pa-Rac1 expressing animals had marked increases in regenerative axons that remained at the DREZ (Figure 6I). This is in stark contrast with the exposed/photoactivated pa-Rac1 expressing animals, which resulted in significantly more axonal retractions (Figure 6I). This indicates that cell-specific photoactivated Rac1 not only markedly diminishes invadopodia stability but correspondingly hinders regenerative potential.

In addition to these new results, we also added to the limitation section that we cannot measure re-entry directly in these cell-autonomous manipulations. While a growth cone maintaining its position at the DREZ 24 hpi does correlate with regeneration, it does not directly confirm it has re-entered into the CNS; however, axonal retraction indicates failed regeneration.

5. Investigate whether overexpression of secreted netrin from glia or other tissues can prematurely close the regenerative window.

We thank the editor for this comment. Such an experiment could help solidify our hypothesis that changes in Netrin close the regeneration period. For technical reasons, we decided this experiment was outside the scope of the current paper. The overexpression experiment requires us to generate a *netrin* expressing plasmid. We would then need to inject that plasmid into the animal, creating a mosaic animal. The mosaicism is a significant concern in this case. In particular, we expected Netrin to be expressed in glia around the DRG and central axon. Unfortunately, we do not have a promoter that expresses in the DRG ensheathing glia that is not also expressed in the neurons. We, therefore, do not have an approach to specifically upregulate Netrin only in glia. Ideally, we could generate a knockin so that we could overexpress it more reliably, but that is a technical approach that exceeded the revision timeline. Generating a stable line from an overexpression plasmid similarly exceeds the revision window. To address the concern, we added text to the limitation paragraph that states overexpression of Netrin would be an ideal experiment but it is technically unfeasible for the scope of this paper.

Journal Requirements:

If the reviewer comments include a recommendation to cite specific previously published works, please review and evaluate these publications to determine whether they are relevant and should be cited. There is no requirement to cite these works unless the editor has indicated otherwise.

1) We ask that a manuscript source file is provided at Revision. Please upload your manuscript file as a .doc, .docx, .rtf or .tex. If you are providing a .tex file, please upload it under the item type u2018LaTeX Source Fileu2019 and leave your .pdf version as the item type u2018Manuscriptu2019.

We have completed this.

2) Please provide an Author Summary. This should appear in your manuscript between the Abstract (if applicable) and the Introduction, and should be 150-200 words long. The aim should be to make your findings accessible to a wide audience that includes both scientists and non-scientists. Sample summaries can be found on our website under Submission Guidelines: https://journals.plos.org/plosgenetics/s/submission-guidelines#loc-parts-of-a-submission

We have completed this.

3) We noticed that you used the phrase 'data not shown' in the manuscript. We do not allow these references, as the PLOS data access policy requires that all data be either published with the manuscript or made available in a publicly accessible database. Please amend the supplementary material to include the referenced data or remove the references.

This has been removed, thank you for pointing this out.

4) We do not publish any copyright or trademark symbols that usually accompany proprietary names, eg ©, ®, or TM (e.g. next to drug or reagent names). Therefore please remove all instances of trademark/copyright symbols throughout the text, including:

- © on page: 24
- ® on page: 24.

These have been removed. Thank you.

5) We notice that your supplementary Figures are included in the manuscript file. Please remove them and upload them with the file type 'Supporting Information'. Please ensure that each Supporting Information file has a legend listed in the manuscript after the references list.

We have completed this.

6) Some material included in your submission may be copyrighted. According to PLOSu2019s copyright policy, authors who use figures or other material (e.g., graphics, clipart, maps) from another author or copyright holder must demonstrate or obtain permission to publish this material under the Creative Commons Attribution 4.0 International (CC BY 4.0) License used by PLOS journals. Please closely review the details of PLOSu2019s copyright requirements here: PLOS Licenses and Copyright. If you need to request permissions from a copyright holder, you may use PLOS's Copyright Content Permission form.
Please respond directly to this email and provide any known details concerning your material's license terms and permissions required for reuse, even if you have not yet obtained copyright permissions or are unsure of your material's copyright compatibility. Once you have responded and addressed all other outstanding technical requirements, you may resubmit your manuscript within Editorial Manager.
Potential Copyright Issues:
i) Figures 7B, and 8B. Please confirm whether you drew the images / clip-art within the figure panels by hand. If you did not draw the images, please provide (a) a link to the source of the images or icons and their license / terms of use; or (b) written permission from the copyright holder to publish the images or icons under our CC BY 4.0 license. Alternatively, you may replace the images with open source alternatives. See these open source resources you may use to replace images / clip-art:
- https://commons.wikimedia.org
- https://openclipart.org/.

Our lab owns these depictions of the zebrafish spinal cord, DRG and central axon, and representation of serial axotomies.

7) We note that your Data Availability Statement is currently as follows: "All data related to this manuscript is available in the enclosed material.". Please confirm at this time whether or not your submission contains all raw data required to replicate the results of your study. Authors must share the “minimal data set” for their submission. PLOS defines the minimal data set to consist of the data required to replicate all study findings reported in the article, as well as related metadata and methods (https://journals.plos.org/plosone/s/data-availability#loc-minimal-data-set-definition).
For example, authors should submit the following data:
1) The values behind the means, standard deviations and other measures reported;

2) The values used to build graphs;
3) The points extracted from images for analysis..

All the relevant raw data and minimal data set information has been included for each figure and supplementary figure. See “Hammer2025-RawData-RegenerativePeriod.xlsx”.

Authors do not need to submit their entire data set if only a portion of the data was used in the reported study.
If your submission does not contain these data, please either upload them as Supporting Information files or deposit them to a stable, public repository and provide us with the relevant URLs, DOIs, or accession numbers. For a list of recommended repositories, please see https://journals.plos.org/plosone/s/recommended-repositories.
If there are ethical or legal restrictions on sharing a de-identified data set, please explain them in detail (e.g., data contain potentially sensitive information, data are owned by a third-party organization, etc.) and who has imposed them (e.g., an ethics committee). Please also provide contact information for a data access committee, ethics committee, or other institutional body to which data requests may be sent. If data are owned by a third party, please indicate how others may request data access.

8) Please amend your detailed Financial Disclosure statement. This is published with the article. It must therefore be completed in full sentences and contain the exact wording you wish to be published.
1) State what role the funders took in the study. If the funders had no role in your study, please state: "The funders had no role in study design, data collection and analysis, decision to publish, or preparation of the manuscript."
2) If any authors received a salary from any of your funders, please state which authors and which funders..
If you did not receive any funding for this study, please simply state: u201cThe authors received no specific funding for this work.u201d

This was completed as requested.

Reviewers' comments:

Reviewer's Responses to Questions

Comments to the Authors:
Please note here if the review is uploaded as an attachment.

Reviewer #1: The authors of this manuscript investigate DRG central axon regeneration after severing, and specifically the mechanisms that close the temporal window during which regeneration can take place. They find that zebrafish larvae can regenerate severed DRG central axons on day 2 of development, but lose that ability on day 3. Using a combination of genetic and pharmacological approaches, they provide evidence that the regeneration window closes because netrin signaling, most likely from adjacent glial cells, prevents appropriate formation of the invadopodia required by severed DRG central axons to enter the spinal cord at the dorsal root entry zone. They examined steps in the netrin signaling pathway and showed that netrin levels increase in glia in the path of regenerating axons at the time when regenerative ability is diminishing, and that expression of the DCC netrin receptor in DRG neurons also increases in the same time frame. Surprisingly, they found that overexpressing DCC in these neurons at a time when the regenerative window is still open does not prematurely close the regeneration window. They did not test whether overexpressing netrin in glia would prematurely close the regenerative window because glia do not reorganize until 3 days. However, since netrin is a secreted molecule, this seems like a missed opportunity to address one prediction of their netrin signaling hypothesis by misexpressing netrin in other cell types, or implanting netrin expressing beads. Instead, they targeted netrin signaling downstream of DCC in dcc heterozygotes, using agonists and antagonists of cAMP and Rac1 and found that upregulation of this pathway decreases and down regulation of this pathway promotes invadopodia persistence at the dorsal root entry zone, and that this occurs through DCC acting within DRG neurons. They also found that antagonizing DCC signaling enabled DRG axons to regenerate following closure of the regeneration window, as assayed by calcium responses to cold water sensory stimuli and resulting behavioral responses that require intact DRG to CNS circuitry.

Thank you for your constructive review. We appreciate how your comments have improved the paper.

The study follows nicely from previous work in the Smith lab. It is generally well done and well presented, although there are a few issues that remain to be addressed.

1) It would be very helpful to clarify which of the processes described in the Introduction (lines 72-84) are referring to mammals, as at the beginning of the paragraph, and which are referring to zebrafish, as at the end of the paragraph. More specifically, are the invadopodia studies cited only from zebrafish? In the following paragraph it would also be useful to state explicitly that this work is from zebrafish.

Thank you for this insightful comment. We have addressed this concern by changing and rewording the sentences to make our references between zebrafish and other model organisms more clear.

“For example, it has long been understood that regeneration of mammalian centrally-projecting axons are inhibited by glial-derived signals and physical scars…”

“Unlike in development, studies in zebrafish demonstrate that regenerating central axons do not form stable invadopodia at the invasion site and thus fail to re-enter and reconnect…”

2) This is a complicated study with many moving parts. It would be really helpful to have a graphical abstract or a summary diagram that puts all the pieces together so that readers can see the entire context, including diagramatic representations of the treatments used to assess the role of DCC downstream components.

We have created a graphical abstract/summary diagram to improve clarity of our model and proposed mechanisms.Thank you.

3) How many DRG neurons are present per DRG at the time of central axon severing in this study? In movie 6 it looks like there are at least two axons and that they go in different directions and perhaps do different things. Do all of the DRGs used in this study have the same number of neurons? Do all of the neurons within a DRG develop within the same time frame? Or are there pioneering neurons and ones that develop later? Do all of the axons from neurons within an individual DRG follow a similar regenerative path? My understanding is that zebrafish DRGs add neurons progressively. I realize that the authors have only investigated the first few days of development, but they might think about, and perhaps discuss, the regenerative capacity of DRG neurons that are added later. In other words, does the regenerative window close early so that only the first-developing DRG neurons can send central axons into the CNS following injury, or is there some kind of sliding window so that later-developing DRG neurons are also able to regenerate during later windows?

The number of DRG neurons is not always clear because they are often condensed and tightly grouped. In general, we observe that DRG consists of 1-3 neurons at the time frames we are doing these analyses. As the animals continue to develop this number increases, but again, it is not always clear without specifically investigating this with various transgenic lines due to their compact nature. To date, we have not observed a trend between DRG soma number or morphology (condensed vs beads on a string) and regenerative capacity.

You are correct in pointing out that there are instances when two central axon projections extend from a single DRG during regeneration. For Movie S6 in particular, one (initial) projection navigates toward the DREZ and then a secondary axon extends later and navigates away from the DREZ. In this movie, the secondary axon goes out of focus during the timelapse, which makes our ability to analyze it properly (consistent with others) impossible, but generally we see that these secondary axons are more transient and seem to be predominantly exploratory.

Furthermore, these secondary projections rarely display the same dynamics as the initial regenerative projection in terms of the balance of filopodia/invadopodia formation. Although interesting, these aberrantly exploring axonal extensions typically retract quickly, and investigating the dynamics of such a projection is outside of the scope of this study.

You also bring up an interesting concept regarding regeneration in nascent sensory neurons that arise later in development, but in our investigation we have yet to see a DRG that did not regenerate the central axon within the first 24 hours post-injury but then later had a fully grown central axon. After the initial regenerative axonal projection fails to re-enter the DREZ they usually retract back to the soma permanently; to date, we have yet to see a secondary wave of axonal projection occur beyond this 24 hpi window. The nascent central axons that develop later within/along the pioneer central bundle seem to use the pioneer axon as a guide and navigate along it to enter into the CNS, rather than going through their own invasion process. This explains why our proposed mechanism of regenerative period closure might occur so quickly after pioneer axon development, because nascent axons within the bundle do not require structures like invadopodia to enter into the CNS. This suggests that the addition of new axons within a given dorsal root is reliant on the existence of the pioneer axon, which also emphasizes the importance of regenerating these axons quickly after injury, particularly in young individuals that continue to develop new and heterogeneous somatosensory connections across the DREZ.

4) It would be very helpful to mark where the cut occurs on at least the first figure showing the axon severing.

This is a helpful comment to guide the readers. We have addressed this by adding a circle onto the pre-injury images of Figure 1B to indicate where the axotomy was targeted specifically. We consistently injure the axons at the midway point of the central axon as this better avoids potential injuries to the dorsal longitudinal fasciculus and neuronal soma. Rather than add this to each pre-injury image in the rest of our figures we added this detail to our methods section "Axotomy and glial ablations".

"To axotomize the centrally-projecting DRG axons, the axon's widest section at the midway point between the DREZ and DRG was put in focus…"

5) Figures and figure panels are not all in order (eg some later panels are described before earlier panels of the same figure). And some panels don't seem to be mentioned in the text.

Thank you for pointing this out. We now introduce figure panels so they are in order. We have also made sure to reference each figure panel in the text.

6) Many of the figure legends should provide more description. For example, in Figure 3F, what do DRG 3, DRG 4, etc. signify?

Thank you for bringing this to our attention. DRG # represent the numerical value of the DRG from the anterior (head) end of the animal. DRG 1 would be the anterior most DRG. We have addressed this in the figure legend of Figure 3.

“(DRG number represents the position of the DRG from anterior to posterior. Anterior-most DRG is 1.)”

7) It’s difficult to compare Figures 5B and 5C since they were made with different graphing formats.

This comment highlighted that we needed to be more clear in the manuscript regarding these figures. We have edited the graphs to have the same graphing format. We also clarified the differences in these graphs in the text by better describing the purpose of these analyses and why each addresses important separate questions.

“...displayed the distribution and frequency of all actin peak durations and compared invadopodia durations throughout the entire navigation process, including at the DREZ, between groups (Figure 5C). These results demonstrated that the stabilizing effects on invadopodia were present at the DREZ (Figure 5B) and not throughout the entire navigation process (Figure 5C).”

8) In Figure 5F, why are the cortactin only IHC panels (two on the right) offset from the others? Is that the same spot as in the second panel from the left? If so, why are they not aligned in all of the panels? If not, what are we seeing?

We appreciate this comment. We have adjusted these images to be better aligned. However, it is worth noting that Cortactin is localized at the distal portion of the invadopodia/growth cone, so it overlaps with a smaller portion of the Lifeact-GFP growth cone.

9) What is the rationale for cutting DRG axons from multiple segments in the behavioral experiments?

This is an important question and something we have adjusted to make more clear in the text. For the behavioral assay the axotomy of a single DRG is not enough to cause a discernable difference in the shivering phenotype. To get an accurate depiction of this shivering phenotype across treatment groups, we perform axotomies to multiple consecutive segments. This makes the shivering phenotype quantifiably clear when an animal had higher rates of central axon regeneration compared to ones that had low regeneration. The decision of performing 8 series axotomies was based on previous works in our lab in which a similar post-regeneration analysis was completed.

10) In the discussion the authors try to draw some distinctions between development and regeneration. However, they don’t carefully define the developmental time frame they focus on, and therefore some of their language seems confusing.

Thank you for this suggestion. We have addressed this by adding more clarification in the text.

“Recent mounting evidence from regenerative studies in mice and zebrafish indicate that glial cells surrounding the DRG undergo distinct changes in response to central axon injury, not only in their organization but in their own expression profiles that negatively impact DRG central axon regeneration[39,40,54].”

“It has long been established that during development of the CNS, radial glia and astrocytes manipulate the location of molecules such as Slits, Netrins, and Semaphorins to influence axon pathfinding in the brain and spinal cord90–95.”

11) There are a number of instances in the manuscript where the subject and verb do not agree. And there are some typos that warrant spell checking.

Thank you, we have completed a detailed review and checked for proper grammar and spelling.

Reviewer #2: The manuscript by Hammer and Smith examines regeneration of the central axons of DRG/somatosensory neurons following laser axotomy in the larval zebrafish spinal cord. The work uses a combination of genetics, chemical genetics and live imaging to investigate the role of glia and a DCC signaling axis -- which previously described by the authors as functioning during the development of this system (Kikel-Coury et al., 2021) -- in DRG axon regeneration.

The work is novel and will be of interest to researchers in the neuroscience and regeneration fields. While the genetic and epistatic experiments are strengths of the manuscript, as discussed below, there are several other areas that should be strengthened/revised prior to publication.

Thank you for your kind remarks and thoughtful suggestions.

Major comments
----

* Although the authors claim that the photoablation system used does not cause excessive damage to surrounding tissue, it was difficult to assess this claim. For example in Fig. 1D there is no control image to compare against. And in Movie S1 there appear to be: 1) examples of cell death; 2) CNS axon regeneration across the lesion site; and 3) increased sox10+ cells in the lesioned area following ablation (all of which could result from non-specific laser damage). As there is not a control video presented here it is unclear whether these are also occurring in the absence of photoablation.

Thank you for giving us an opportunity to address this concern. We have now included a control image to demonstrate the underlying cell damage that occurs with the laser injury.

We would like to note that from volume EM analysis, we have visualized that the DRG central axon is directly adjacent to the glial limitans boundary. The proximity of the axon to the boundary makes it possible we are also perturbing the glial limitans. To avoid this, we focus the laser a z-position away from the boundary, but we cannot rule out that the glial limitans could be slightly injured. In many cases, the fluorescence of the glial limitans marker is restored quickly, more consistent with photobleaching than injury. We have added pre-injury images of the DRG and glial limitans to Figure 1D to better represent this case. For transparency, we kept Movie S1 in the manuscript because it shows one of our most severe examples of such damage or photobleaching. This is a rare occurrence as you can see in our other figures and other movies. In these instances, if the animal survives, we quantify our results to include neuronal apoptosis when applicable, such as in re-entry quantifications for example (Figure 4). Therefore, if the injury was more extensive than normal and neuronal death was observed we do not misrepresent the post-injury results as merely failed regeneration which would belie the results, nor do we simply exclude that replicate, but rather include it as its own category. However, we have seen numerous instances where near perfect axotomies resulted in neuronal death while apparently more severe injuries did not. Albeit rare, this is a variable that exists with laser-induced injury models, and being that we account for it in our analysis, we feel confident

that it is not favorably influencing our results in either direction. With the addition of pre-injury/control images, we feel that Figure 1D does well to represent the normal results of central axon axotomy, with a mildly photobleached injury site immediately following the injury.

* The involvement of glia in the regenerative response remains speculative. The authors propose that glia reorganize following axotomy at 3 dpf but not 2 dpf and that they inhibit regeneration at 3 dpf. However, there are several issues with the analysis. First, as shown in Fig. 2A it appears that the main glial reorganization is between 2 and 3 dpf not after the 3 dpf axotomy as claimed. To address this, in Figure 2C, the authors should compare the two glial values to each other. They should also calculate the relative change in glial position over the two experimental windows (t140-t0) to address the relative amount of reorganization. Second, the glia ablation experiment is lacking an essential control, which is an ablation with the same laser exposure as shown in 2D (targeted outside the glia). Without this, it is unclear whether the increased axon regeneration is due to loss of the glia or increased laser damage (which could cause increased ROS. See e.g., Rieger S, Sagasti A (2011) DOI: 10.1371/journal.pbio.1000621). Third, a limitation of the glial reorganization analysis is that they are only observing nuclear position and not membrane morphology. This could be mentioned in the “limitations” subsection of the discussion.

We appreciate you pointing out these concerns. If I understand your concern correctly, you are concerned with a claim that central axon injury is solely inducing glial re-organization in only 3 dpf animals. I think our text did not clearly highlight that we believe our data demonstrates that glial re-organization is an innate process of development that is predominantly present in 3 dpf animals - coinciding with regenerative period closure. To provide experimental evidence of this, we analyzed glial nuclei migration distances for 140 timepoints (~11.6 hrs) after central axon re-extension begins so that we can deduce if glial re-organization: 1) continues in 3 dpf animals after injury and 2) could be differentially affecting regeneration between 2 dpf animals (when the regenerative period is open) and 3 dpf animals (when the period is markedly closed). The distinctions made from this analysis were important to set our examination of regenerative capacity after glial ablation. This combined with our quantification of *netrin1b* expression being elevated dorsal to the DRG (directly in the trajectory of the regenerative axon) in 3 dpf animals corresponds to increased DCC signaling and an overt downregulation of invadopodia at this developmental stage.

Your second comment is a great addition to the paper. We have addressed it by performing mock injuries/ablations outside of the glia and central axon (added to Figure 2A-C) and measured that glia did not differentially migrate from the mock ablations by 16 hours post mock ablation (Figure 2C). We have also calculated the migration distances of glia from t0-t140 per your excellent suggestion, which highlights the migration distances occurring during regeneration between these ages and injured status (Figure 2D). These results indicate that rather than being dependent on axotomy, glial re-organization is likely an innate developmental process.

Additionally, we have added a mock glial ablation experiment prior to central axon axotomy to investigate if photodamage in general affects regeneration. We observe that general photodamage outside of the glial does not improve regeneration capacity compared to glial ablation (Figure 2F).

We have addressed your third concern here by adding text that states our analysis is limited to nuclear localizations rather than membrane in our limitations section.

"Our quantification of glial re-organization were completed by tracking the migration of glial cell nuclei which is indicative but not resolute in establishing the scale of migration for glia."

* The cell-specific manipulations of DCC and Rac1 are based on the expression of UAS-driven constructs in the sox10:Gal4 line. Because sox10 is expressed in several cell types (somatosensory neurons, surrounding glia, neural crest precursors) in the area (Zhu et al. (2019) DOI: 10.1016/j.cell.2019.08.001), in the absence of a more specific driver, the authors should temper/clarify their language around the cell specificity claims.

We appreciate this comment and agree with the suggestion. We were particularly careful to only include analyses to the presence of puncta inside the neuron/axon rather than in the bordering glial cells. Also, we made sure to only say DRG cells rather than DRG neurons. Nevertheless, per your suggestion we have tempered our claims.

"Since a specific driver that expresses reliably in DRG neurons vs glia has not been identified in zebrafish…"

"…this experimental paradigm tested the specific requirement in DRG cells."

"…we injected a photoactivatable Rac1 construct (UAS:pa-Rac1-mCherry) into Tg(sox10:Gal4+myl7:GFP);(UAS:LifeAct-GFP) embryos, allowing us to again manipulate the signaling cascade in DRG cells[69]."

* Statistics. The numerous P-values displayed on several of the plots were distracting (e.g., Figs 7D,E, 8D) – the authors may consider moving some of this reporting information into the supplement to focus on the data display. Were the statistical tests corrected for multiple comparisons? The use of the "Fisher's Exact t-test" was curious for two reasons: 1) it is not a t-test; and 2) it is only for 2x2 tables but appears to have been used in cases with three categories (e.g., Figs 1E, 4B).

Thank you for your question. We specify that the Fisher's Exact test was used to evaluate differences in re-entry versus failed re-entry rates between groups, which is the primary comparison here. The incorporation of neuron apoptosis into the graph was to maintain transparency of our results. Being that the scope of this study, particularly in figures scoring 24 hpi outcomes between groups, were concerned with the proportion of regeneration and failed regeneration rates between treatment/genetic condition groups, Fisher's Exact test is ideal. We

have minimized the number of p-values on the graphs to be more digestible, and make all p-value comparisons available in our Raw Data file for each figure. We removed “t-test” from the description. We also highlighted that the Fisher’s Exact test was comparing re-entry vs failed re-entry (or synchronized vs delayed neuronal activity for the calcium imaging assay) in the text and legends to improve clarity.

“Comparisons were made on the basis of central axon re-entry and failed re-entry using Fisher’s Exact test between treatment groups and DMSO vehicle control.”

Minor comments
----

* A developmentally regulated diminished capacity for skin reinnervation was previously described for larval somatosensory neurons by O’Brien et al. (2009) DOI: 10.1016/j.cub.2009.10.051. It would be important to contextualize the current results relative to this previous study.

Thanks for bringing this to our attention. The following sentence was added to address this comment: “In zebrafish, somatosensory neuron regeneration of axons that innervate the skin diminishes with age.” and cited the O’Brien paper.

* It would be helpful to contextualize the results of the first results section relative to known events that are happening at 2 vs 3 dpf for zebrafish DRG neurons. For example, when does growth of the central axon begin/terminate?

Thank you for this, details have been added to the first results section to contextualize our results.

“Zebrafish DRG pioneer axons extend, invade and bifurcate in the spinal cord between 40-48 hpf, while also adding additional axons in the nerve after the initial pioneer axon invades.”

* Figure legends do not indicate what the error bars represent (standard deviation, standard error of the mean, etc).

These details have been added to the legends and text. Thank you.

* Figure 1 and lines 110-116. The panels are referred to out of order in the text. Figure 1B is not mentioned in the text.

This has been adjusted, thank you.

* Figure 1A. Consider adding labels or text to indicate the axes (e.g., Dorsal/Ventral)

This has been completed. Thank you.

* Figure 1C and Figure 4D,F. Missing scale bars and hard to see – consider adding a higher magnification inset.

We are unable to insert scale bars into these sideview images.

* Figure 3C,D. Descriptions are reversed in figure legend.

This has been fixed, thank you for pointing this out.

* Figure 3D and lines 154-156. Netrin looks like it is broadly increased rather than specifically dorsal. Considering quantifying and comparing the lateral and ventral domains.

This is important so thank you for pointing this out so we can make it more clear in the text. Figure 3D display a z-cropped max projection of the spinal cord floor plate, so that the entirety of the contents within the spinal floor plate are stacked into one image while excluding tissue outside of this cropped z-stack. Therefore, parts, not the entirety, of the ventral DRG neurons were included into the representative z-stack image as a byproduct of the crop. Thus, the apparent differences you point out around the DRG neuron between 2 and 3 day animals were actually due to the crop itself and are not representative of the expression around the DRG. The expression in the spinal floor plate is what we are attempting to display. The figure legend for Figure 3D now reads "Representative z-cropped max projection images" to better clarify this. Expression of *netrin1b* ventral to the DRG is not addressed given that the study focuses on DRG central axon regeneration and the dorsal re-organization of peripheral, Netrin-expressing glia around those axons.

* Line 158. It is unclear how the "midpoint of regeneration" was defined.

This comment reminded us we need to be more clear in the text. Midpoint of regeneration refers to a temporal period when axons are approximately 50% through the regeneration process. Referring to the midpoint is the midpoint in the regenerative process, in 2 dpf animals the central axon regenerates readily and often re-entered the DREZ ~8 hpi, while 3 dpf animals are delayed, often going through several extension/retractions cycles during its navigation to the DREZ, and when they do re-enter it was ~20 hpi. This makes 4 hpi in 2 dpf animals and 10 hpi for 3 dpf animals the midpoint of regeneration. We have tried to make appropriate adjustments to make this more clear.

"...these times represent the respective midpoint of regeneration attempts (when axons are approximately 50% through the regenerative process) at these ages."

* Figure 3B. A small schematic may be helpful for explaining the experimental design.

Thank you for bringing this to our attention. We added white dashed tracings to the images of Figure 3A to represent how the HCR analysis for Netrin was completed. This is now clarified in the figure legend as well.

“White dashed tracings indicate how the dorsal expression of *netrin1b* was obtained – traced from the top of the DRG to the DREZ signified by the dorsal longitudinal fasciculus.”

* Lines 173-174. The involvement of netrin1b remains speculative and the text on L173-4 “both dcc and netrin1b are organized in the tissue to close the regenerative period at 3 dpf.” was unclear/imprecise.

This has been amended to improve clarity.

“These findings, coupled with our conclusion that glia dorsally migrate/re-organize and therefore increase the glial expression of *netrin1b* dorsal to the DRG, support a model in which both *dcc* and *netrin1b* are spatiotemporally organized in the tissue to close the regenerative period at 3 dpf.”

In the text, the authors should state: What is the nature of the ZM130198 allele? Why was this allele chosen relative to other available dcc alleles?

Thank you for pointing out this detail. We have added the appropriate text as suggested.

“This ZM130198 allele incorporates a 5.2 kb retrotransposon insertion into the 5’ UTR of dcc (106 bp upstream of the start codon) and was selected because it results in >90% reduction of dcc mRNA in homozygous mutant zebrafish”

* Figure 4G,H. What was the treatment/background for these panels?

Thanks for catching that lack of clarity (Failed regeneration (DMSO), regeneration (rp-cAMP). This has been added to the Figure 4G and 4H legends.

* Lines 226-228. The rationale for suspecting a failure in invadopodia formation was unclear. Seems like the movies/images don’t support a failure at the DREZ but earlier. Consider revising the text to better set up logic/hypothesis

We appreciate you pointing this out as it is an important component.. We have added additional information to better set up our hypothesis.

“The DCC-cAMP-Rac1 signaling pathway has been shown to spatiotemporally downregulate invadopodia formations during pioneer central axon development, until the developmental growth cone reaches the DREZ and invasion events are required[43]. Based on our observations that glial re-organization and increased netrin presentation dorsal to the DRG coincides with regenerative period closure, we suspected that DCC signaling may become inherently dysregulated and continue to regulate cytoskeletal dynamics in the growth cone in 3 dpf animals.”

* Figure 5A and Figure 6. Typo on y-axis labels “Intesnity”.

Thank you, these have all been fixed.

* Figure 5B,C. What are the n values?

This is described in the figure legends or text.

* Figure 7C. What does the “lz-score” label signify?

The vertical line in front of “z-score” indicates the vertical increase in the z-score line graphs that correspond a change of 2.0, which was our indicator if a neuron underwent neuronal activation or not. We have added that detail to the figure legend.

“The vertical line in front of “z-score = 2.0” indicates the vertical increase in the individual traces (lines) needed to reach a z-score of 2.0 and be considered an activated neuron.”

* Supplemental movies lack scale bars.

Thank you, we have added this to all movies.

* Reference 61 is a duplicate of 45 and reference 70 is incomplete.

This has been amended.

Reviewer #3: This manuscript by Hammer and Smith interrogates the closure of a window of regenerative plasticity in DRG neurons of Zebrafish. Understanding the molecular mechanisms that govern the switch from a plastic state during development that is conducive to regenerative and growth, to a 'post-developmental' state that is not permissive is a key question in neurobiology. Understanding how this state change is regulated will be important to develop therapies for neurodevelopmental disorders as well as disease and injuries to the nervous system.

The authors characterise an in vivo model of regeneration using the DRG neurons of zebrafish and establish that they undergo robust regeneration that rapidly declines over a 24hr window between 2dpf and 3dpf. They identify that glial cell populations also reorganise during this time window and identify netrin signalling as a key regulator of this process, with glial cells the likely source. Using a combination of transgenic lines and pharmacological manipulations they present evidence that netrin functions via DCC>cAMP>Rac1 in DRG neurons to regulate regenerative potential and the closing of this regenerative period.

The authors using live imaging to characterise the development of distinct invadopodia and actin dynamics that correlate with regenerative potential that are regulated by this signalling axis. The show that regenerative potential correlates with functional recovery and that manipulating the netrin/DCC/rac pathway can restore both spinal motorneuron activity and behaviour following injury.

This is an important and interesting study that will be of interest to the field of axonal regeneration, glial cell biology and axonal guidance.

Thank you for your kind remarks and suggestions to improve the manuscript.

There are several elements that I think can be refined to improve the manuscript and solidify the main findings.

Main points:

1. The decline in regenerative potential is characterised as a window, that can be manipulated with DCC/cAMP/rac1 signalling to be 're-opened'. I don't think the data fully supports this notion. First, in Figure 1 the regenerative decline is characterised across 2dpf, 3dpf and 5dpf. All other data in which the window is re-opened is only done at 3dpf. This supports the notion that the decline is prevented until after 3dpf. But does not provide any information about any regenerative potential after 3dpf. This is important to distinguish if these animals have no decline in potential, or a delay. Moreover, to support the notion that a window is 'reopened' – something needs to close. Ie. a condition in which 3dpf there is limited regeneration and then a manipulation to restore regeneration in 5dpf. This is a major claim of the manuscript. I think the language needs to be revisited if the data is not supporting this concept.

Thank you for this insightful comment. We addressed this comment with your suggested experiment. We completed single central axon injuries in 5 dpf treated with rp-cAMP and

NSC23766 (the cAMP and Rac1 antagonists) in combination. This combination was selected based on data in Figure 4 that demonstrated the highest amount of regeneration in the double manipulation. We also could selectively add the molecules at the 5 dpf, allowing any possible regeneration window to completely close before testing if we could re-open it and promote regeneration. From these new results (Figure 4I-K) we see a significant increase in central axon regeneration back into the CNS, which is in stark contrast to 5 dpf DMSO control treated animals. These findings are consistent with the hypothesis that antagonistic manipulation of the DCC-cAMP-Rac1 signaling pathway re-opens the regenerative period.

2. The quantification of glia between 2 and 3 dpf is very interesting and in the images and videos presented there is a stark difference. I would be very interested to see the localisation of glial cells at these ages in uninjured animals to further characterise this relationship. At 3dpf it appears that some glia flank the base of the central axon. Is this a response to injury, or the normal cell position at this age? Furthermore, what is the glia phenotype in the DCC/cAMP/Rac1 manipulations? Do they still localise around the axon in animal where the regenerative potential is regained?

Thank you for this question, we found it very intriguing and it led to important additions to the study. According to what we have seen in our experiments it seems that it is normal, regardless of injury, for glial nuclei to flank the base of the central axon (Smith et al. 2017., Nichols et. al. 2018, Nichols and Smith 2019). However, after the 3 dpf mark glial nuclei also begin to dorsally migrate and begin populating the border of the central axon as well as the base of the axon (Smith et. al. 2017).

To address your questions as well as other comments from the other reviewers, we performed mock injuries/ablations to the outside of glial nuclei and central axon to see if glial re-organization is dependent on injury in 3 dpf animals (bottom of Figure 2A-C). We found that it did not cause a discernible difference in re-organization in 3 dpf animals 16 hours post-mock ablation compared to injured 3 dpf animals (Figure 2D).

To test if antagonistic manipulations are affecting glial re-organization, we also did this analysis in *dcc*$^{+/-}$ animals, wt animals, as well as rp-cAMP treated wt animals and NSC23766 treated wt animals, each analyzed 12 hpi. We also did this in uninjured animals at the 72 hpf (3 dpf) mark . This analysis revealed no change from treatment in terms of the re-organization of glial nuclei (Figure 4L and S1G-I). This suggests that antagonistic manipulations are unlikely to impact glial re-organization. Additionally, we performed an analysis of total migration distance of the glial nuclei between t0 and t140 between 2 and 3 dpf animals as well 3 dpf animals that underwent mock injury/ablation (Figure 2D). Collectively, we think these additional experiments and analyses address your concerns, highlighting that dorsal re-organization of peripheral glia is innately and predominantly present in 3 dpf animals and not 2 dpf animals. Collectively, these are addressed in our additional data added to Figure 2, and Figure 4 & S1. These data demonstrate that, in the presence or absence of central axon injury, the glial cells continue to be localized dorsal to the DRG with highly comparable distances from the dorsal border of the DRG.

3. The experiments using cell-specific manipulations of DCC and Rac1 are only characterised with invadopodia imaging and functional assays. Demonstrating that these manipulations alter the regeneration phenotype would solidify this data.

This was a great comment. Addressing this with our conventional re-entry analysis (orthogonal displacement quantification) which utilizes the *gfap:mCherry*$^{+}$ glial boundary of the spinal cord could not be used because the *dcc-tdTomato* and *pa-Rac1-mCherry* would be lost in the *gfap:mCherry*$^{+}$ spinal boundary present in the background. Additionally, distinguishing between mCherry signal from the glial limitans versus the fused proteins makes such analysis technically unfeasible. Therefore, to address this, we revisited the LifeAct timelapses of Figure 6 and quantified whether the regenerative axon maintained its position at the DREZ at the end of the timelapse or retracted (Figure 6E & 6I). We quantified that *dcc*$^{+/-}$ animals expressing *dcc*-tdTomato had increased rate of retraction by the end of the 24 hour timelapse (Figure 6E). This was significantly different from the *dcc*$^{+/-}$ animals injected with *dcc*-tdTomato and treated with rp-cAMP, which functions downstream of DCC in the cascade. Further, we repeated this analysis with pa-Rac1 injected animals, in which we quantified that photoactivated pa-Rac1^{+} DRG have significantly more retraction compared to unactivated (unexposed to 445 nm light) animals despite both groups expressing pa-Rac1 and treated with rp-cAMP (Figure 6I). This addresses if cell-specific manipulations of DCC and Rac1 are not only affecting invadopodia but also the regenerative outcomes of the axons. While not resolute in terms of scoring re-entry, the rate at which axons remained at the DREZ versus retracted by 24 hpi are indicators of regenerative potential.

Minor points:

1. A scheme of the orthogonal view shown in Figure 1C (similar to the inset in Figure 1A) might help with understanding the anatomy of the phenotype and how regeneration was scored. It took me a little while to wrap my head around this the first time I read it.

If we understand this comment correctly, you would like a schematic for Figure 1C to relate to how we are scoring re-entry (orthogonal displacement quantifications). We represent both the anatomical configuration of the tissue and a schematic for orthogonal displacement quantifications in Figure 1A. This displays a line drawn across the point where the distal tip of the axon and the spinal cord boundary meet, representing where we are measuring the fluorescent profile of both the axon and spinal cord to quantify if the axon is inside (left) of the spinal boundary – entered – or if the axon is outside (right) of the spinal boundary – not entered. We have added further clarity in the text to better highlight what Figure 1A is exemplifying and its relationship with the results in Figure 1C.

“We assayed re-invasion and re-entry with a published analysis protocol, orthogonal displacement quantifications, where the dorsal tip (growth cone) of the GFP+ regenerating axon, if present, was located in the orthogonal image relative to the mCherry+ glial limitans (Figure 1A)[43]. Plotting the fluorescent intensity profile at the tip of the GFP+ axon allowed us to

determine if the regenerated axon was medial or lateral to the mCherry+ glial limitans (Figure 1C)."

2. Many of the figures do not recapitulate the detail captured in the live movies. In particular, the lifeact videos are stunning – with lots of information and dynamics that are not represented in the static figure. This is challenging for all live imaging. But I would recommend revising these figures to try and capture more of the dynamics. The actin traces are useful – but the image panels are very small and it's difficult to see the actin structures in the growth cone. The same is true for the images of regeneration in Figure 1.

We appreciate your comments. These static figures are meant to highlight the key details of the effects from each manipulation. We agree that the movies do well to demonstrate the true effects, but in the interest of space and adequately comparing the key details of the results we are also concerned that inserting additional images for the sake of providing more examples of the dynamic effects could distract the reader from the quantifications, particularly the growth cone actin traced line graphs. However, the movies will be readily available to the reader in the event that readers are interested in the dynamics of cells after regeneration. For Figure 1 the axons retracted at 3 and 5 dpf. The orthogonal displacement quantification in Fig 1C represents the axon's position outside of the DREZ prior to permanently retracting.

3. Related to the above point. It's not entirely clear what primary phenotype is in 3dpf injured control animals vs DCC manipulations. In Figure 1 and Movie S2 it appears that at 3dpf the central axon never really makes it out of the glial surrounding region just dorsal to the injury. But in the lifeact images it appears that the axon extends – but is not stabilised and retracts. These are important details. Especially given the correlative data in relation to glia as a source of netrin to inhibit regeneration.

Thank you for this comment. In control animals, regenerating axons typically extend dynamically until they reach the DREZ. If regeneration fails, the axon will retract, leaving the cell soma without an axon that connects to the CNS. This phenotype is observed in our published paper (Nichols and Smith 2019), as well as in Figures 2A and 4C. In the $dcc^{-/-}$ animals, the axons similarly extend but re-invade the spinal cord. As a result, regenerating axons in *dcc*$^{-/-}$ animals do not retract. Figure 5B also provides information about the axons approaching the DREZ. In all treatment groups, the axons still regenerate to the DREZ. However, treatments that do not induce re-invasion result in retraction. We added the quantification of axons that remain at the DREZ vs retract in the cell-autonomy experiments in Figure 6E and 6I to further clarify this point. We have added text to note that axons that fail to re-invade often retract. We hope this addresses your thoughtful comment.
